# The Albumen of the Blood in Cholera

**Published:** 1866-09

**Authors:** 


### The Albumen of the Blood in Cholera.

The researches of M. Fernaud Papillon on the chemical and physical constitution of the blood in cholera have been published in the *Journal de l'Anatomie*, (No. 2) and deserve the notice of the profession. His observations on the nature of the albumen removed from the blood of cholera patients during the algide period show that the fluid differs materially from the normal albumen. The albumen was separated from the corpuscles by repeated filtrations. The following are the results of M. Papillon's experiments: (1.) This albumen, placed for four days in water, became neither hydrated nor swollen; it remained just as it was when first added, although ordinary albumen is either dissolved or swells up under the same circumstances. (2.) It does not dissolve in potash or soda, even at an elevated temperature, although ordinary albumen is soluble in these reagents, even at ordinary temperatures. (3.) When treated with hydrochloric acid, it slowly dissolves, and the solution, instead of having the usual deep-violet color, is only faintly tinted. (4.) At the ordinary temperatures, common albumen decomposes rapidly a mixture of nitric and sulphuric acids, nitrous vapors being disengaged. Choleraic albumen does not do so at the ordinary temperature. Ordinary albumen is very rapidly dehydrated by sulphuric acid; the choleraic albumen is affected only after a long exposure.—*Lancet.*

